# On Local Convergence Analysis of Inexact Newton Method for Singular Systems of Equations under Majorant Condition

**DOI:** 10.1155/2014/498016

**Published:** 2014-08-28

**Authors:** Fangqin Zhou

**Affiliations:** Department of Mathematics and Physics, Quzhou University, Quzhou 324000, China

## Abstract

We present a local convergence analysis of inexact Newton method for solving singular systems of equations. Under the hypothesis that the derivative of the function associated with the singular systems satisfies a majorant condition, we obtain that the method is well defined and converges. Our analysis provides a clear relationship between the majorant function and the function associated with the singular systems. It also allows us to obtain an estimate of convergence ball for inexact Newton method and some important special cases.

## 1. Introduction

Newton's method and its variations, including the inexact Newton methods, are the most efficient methods known for solving the following nonlinear equation:

(1) $$\begin{array}{c} F\left(x\right)=0, \end{array}$$

where *F* : *Ω* ⊂ *R* ^*n*^ → *R* ^*m*^ is a nonlinear operator with its Fréchet derivative denoted by *DF*; *Ω* is open and convex. In the case *m* = *n*, the inexact Newton method was introduced in [1] denoting any method which, given an initial point **x**_0_, generates the sequence {**x**_*k*_} as follows:

(2) xk+1=xk+sk,  DF(xk)sk=−F(xk)+rk,k=0,1,2,…,

where the residual control **r**_*k*_ satisfies

(3) $$\begin{array}{c} ||r_{k}||\leq \lambda_{k}||F\left(x_{k}\right)||,\;k=0,1,2,\ldots , \end{array}$$

and {*λ*_*k*_} is a sequence of forcing terms such that 0 ≤ *λ*_*k*_ < 1. Let **ζ** be a solution of (1) such that *DF*(**ζ**) is invertible. As shown in [1], if *λ*_*k*_ ≤ *λ* < 1, then, there exists *r* > 0 such that, for any initial guess **x**_0_ ∈ **B**(**ζ**, *r*): = {**x** ∈ *R* ^*n*^ : ||**x** − **ζ**||<*r*}, the sequence {**x**_*k*_} is well defined and converges to a solution **ζ** in the norm ||**y**||_∗_ : = ||*DF*(**ζ**)**y**||, where ||·|| is any norm in *R* ^*n*^. Moreover, the rate of convergence of {**x**_*k*_} to **ζ** is characterized by the rate of convergence of {*λ*_*k*_} to 0. It is worth noting that, in [1], no Lipschitz condition is assumed on the derivative *DF* to prove that {**x**_*k*_} is well defined and linearly converging. However, no estimate of the convergence radius *r* is provided. As pointed out by [2] the result of [1] is difficult to apply due to dependence of the norm ||·||_∗_, which is not computable.

It is clear that the residual control (3) is not affine invariant (see [3] for more details about the affine invariant). To this end, Ypma used in [4] the affine invariant condition of residual control in the form

(4) $$\begin{array}{c} ||DF{\left(x_{k}\right)}^{-1}r_{k}||\leq \lambda_{k}||DF{\left(x_{k}\right)}^{-1}F\left(x_{k}\right)||,\;k=0,1,2,\ldots , \end{array}$$

to study the local convergence of inexact Newton method (2). And the radius of convergent result is also obtained.

To study the local convergence of inexact Newton method and inexact Newton-like method (called inexact methods for short below), Morini presented in [2] the following variation for the residual controls:

(5) $$\begin{array}{c} ||P_{k}r_{k}||\leq \lambda_{k}||P_{k}F\left(x_{k}\right)||,\;k=0,1,2,\ldots , \end{array}$$

where {*P*_*k*_} is a sequence of invertible operators from *R* ^*n*^ to *R* ^*n*^ and {*λ*_*k*_} is the forcing term. If *P*_*k*_ = **I** and *P*_*k*_ = *DF*(**x**_*k*_) for each *k*, (5) reduces to (3) and (4), respectively. Both proposed inexact methods are linearly convergent under Lipschitz condition. It is worth noting that the residual controls (5) are used in iterative methods if preconditioning is applied and lead to a relaxation on the forcing terms. And we also note that the results obtained in [2] cannot make us clearly see how big the radius of the convergence ball is. To this end, Chen and Li [5] obtained the local convergence properties of inexact methods for (1) under weak Lipschitz condition, which was first introduced by Wang in [6] to study the local convergence behavior of Newton's method. The results in [5] easily provide an estimate of convergence ball for the inexact methods. Furthermore, Ferreira and Gonçalves presented in [7] a new local convergence analysis for inexact Newton-like method under the so-called majorant condition.

Recent attentions are focused on the study of finding zeros of singular nonlinear systems by Gauss-Newton's method, which is defined as follows. For a given initial point **x**_0_ ∈ *Ω*, define

(6) $$\begin{array}{c} x_{k+1}=x_{k}-DF{\left(x_{k}\right)}^{†}F\left(x_{k}\right),\;k=0,1,2,\ldots , \end{array}$$

where *DF*(**x**_*k*_)^†^ denotes the Moore-Penrose inverse of the linear operator (or matrix) *DF*(**x**_*k*_). For example, Shub and Smale extended in [8] the Smale point estimate theory (including *α*-theory and *γ*-theory) to Gauss-Newton's methods for underdetermined analytic systems with surjective derivatives. For overdetermined systems, Dedieu and Shub studied in [9] the local linear convergence properties of Gauss-Newton's method for analytic systems with injective derivatives and provided estimates of the radius of convergence balls for Gauss-Newton's method. Dedieu and Kim in [10] generalized the results of both the underdetermined case and the overdetermined case to such case where *DF*(**x**) is of constant rank (not necessarily full rank), which has been improved by some authors in [11–16].

In the last years, some authors have studied the convergence behaviour of inexact versions of Gauss-Newton's method for singular nonlinear systems. For example, Chen [17] employed the ideas of [5] to study the local convergence properties of several inexact Gauss-Newton-type methods where a scaled relative residual control is performed at each iteration under weak Lipschitz conditions. Ferreira et al. presented in their recent paper [18] a local convergence analysis of an inexact version of Gauss-Newton's method for solving nonlinear least squares problems. Moreover, the radii of the convergence balls under the corresponding conditions were estimated in these two papers.

In the present paper, we are interested in the local convergence analysis; that is, based on the information in a neighborhood of a solution of (1), we determine the convergence ball of the method. Following the ideas of [7, 14, 18–20], we will present a new local convergence analysis for inexact Newton's method for solving singular systems with constant rank derivatives under majorant condition. The convergence analysis presented provides a clear relationship between the majorizing function, which relaxes the Lipschitz continuity of the derivative, and the function associated with the nonlinear operator equation. Besides, the results presented here have the conditions and the proof of convergence in quite a simple manner. Moreover, two unrelated previous results pertaining to inexact Newton method are unified, namely, the result for analytical functions and the classical one for functions with Lipschitz derivative.

The rest of this paper is organized as follows. In Section 2, we introduce some preliminary notions and properties of the majorizing function. The main results about the local convergence are stated in Section 3. And finally in Section 4, we prove the local convergence results given in Section 3.

## 2. Notations and Auxiliary Results

For **x** ∈ *R* ^*n*^ and a positive number *r*, throughout the whole paper, we use **B**(**x**, *r*) to stand for the open ball with radius *r* and center **x**, and let $\bar{B(x,r)}$ denote its closure.

Let *A* : *R* ^*n*^ → *R* ^*m*^ be a linear operator (or an *m* × *n* matrix). Recall that an operator (or *n* × *m* matrix) *A* ^†^ : *R* ^*m*^ → *R* ^*n*^ is the Moore-Penrose inverse of *A* if it satisfies the following four equations:

(7) $$\begin{array}{c} A^{†}AA^{†}=A^{†};\;\;AA^{†}A=A;\\ {\left(AA^{†}\right)}^{*}=AA^{†};\;\;{\left(A^{†}A\right)}^{*}=A^{†}A, \end{array}$$

where A* denotes the adjoint of *A*. Let *ker*⁡*A* and im *A* denote the kernel and image of *A*, respectively. For a subspace *E* of *R* ^*n*^, we use Π_*E*_ to denote the projection onto *E*. Then, it is clear that

(8) $$\begin{array}{c} A^{†}A=\Pi_{\ker A^{⊥}},\;\;AA^{†}=\Pi_{\text{im}\;A}. \end{array}$$

In particular, in the case when *A* is full row rank (or equivalently, when *A* is surjective), *AA* ^†^ = **I**_*R*^*m*^_; when *A* is full column rank (or equivalently, when *A* is injective), *A* ^†^ *A* = **I**_*R*^*n*^_.

The following lemma gives a perturbation bound for Moore-Penrose inverse, which is stated in [21].

**Lemma 1 (see [21], Corollary 7.1.1 and Corollary 7.1.2 ).**

Let *A* and *B* be *m* × *n* matrices and let *r* ≤ min⁡{*m*, *n*}. Suppose that rank⁡ *A* = *r*, 1 ≤ rank⁡ *B* ≤ rank⁡ *A*, and ||*A* ^†^||||*B* − *A*||<1. Then, rank⁡ *B* = *r* and

(9) $$\begin{array}{c} ||B^{†}||\leq \frac{||A^{†}||}{1-||A^{†}||||B-A||}. \end{array}$$

Also, we need the following useful lemma about elementary convex analysis.

**Lemma 2 (see [22], Proposition 1.3).**

Let *R* > 0. If *g* : [0, *R*] → *R* is continuously differentiable and convex, then

- (*g*(*t*) − *g*(*τt*))/*t* ≤ (1 − *τ*)*g*′(*t*) for all *t* ∈ (0, *R*) and *τ* ∈ [0,1],
- (*g*(*u*) − *g*(*τu*))/*u* ≤ (*g*(*v*) − *g*(*τv*))/*v* for all *u*, *v* ∈ [0, *R*), *u* < *v*, and 0 ≤ *τ* ≤ 1.

For a positive real *R* ∈ *R* ^+^, let

(10) $$\begin{array}{c} f:\left[0,R\right]\times \left[0,1\right)\times \left[0,1\right)⟶R \end{array}$$

be a continuously differentiable function of three of its arguments and satisfy the following properties:.

- *f*(0, *λ*, *θ*) = 0 and (∂/∂*t*)*f*(*t*, *λ*, *θ*)|_*t*=0_ = −(1 + *λ* + *θ*),
- (∂/∂*t*)*f*(*t*, *λ*, *θ*) is convex and strictly increasing with respect to the argument *t*.

For fixed *λ*, *θ* ∈ [0,1), we write *h*_*λ*,*θ*_(*t*)≜*f*(*t*, *λ*, *θ*) for short below. Then the above two properties can be restated as follows:

(iii) * h*_*λ*,*θ*_(0) = 0 and *h*_*λ*,*θ*_′(0) = −(1 + *λ* + *θ*),
(iv) * h*_*λ*,*θ*_′(*t*) is convex and strictly increasing.

Set

(11) $$\begin{array}{c} \xi ∶=\sup\left\{t\in \left[0,R\right):h_{0,0}^{\prime }\left(t\right)<0\right\}, \end{array}$$

(12) $$\begin{array}{c} \rho ∶=\sup\left\{t\in \left[0,\xi \right):\frac{h_{\lambda ,\theta }\left(t\right)}{h_{0,0}^{\prime }\left(t\right)}-t<t\right\}, \end{array}$$

(13) $$\begin{array}{c} \sigma ∶=\sup\left\{t\in \left[0,R\right):B\left(\zeta ,t\right)\subset \Omega \right\}. \end{array}$$

The next two lemmas show that the constants *ξ* and *ρ* defined in (11) and (12), respectively, are positive.

**Lemma 3 .**

The constant *ξ* defined in (11) is positive and *t* − *h*_*λ*,*θ*_(*t*)/*h*_0,0_′(*t*) < 0 for all *t* ∈ (0, *ξ*).

**Proof**

Since *h*_0,0_′(0) = −1, there exists *δ* > 0 such that *h*_0,0_′(*t*) < 0 for all *t* ∈ (0, *δ*). Then, we get *ξ* ≥ *δ*(>0). It remains to show that *t* − *h*_*λ*,*θ*_(*t*)/*h*_0,0_′(*t*) < 0 for all *t* ∈ (0, *ξ*). Because *h*_*λ*,*θ*_′ is strictly increasing, *h*_*λ*,*θ*_ is strictly convex. It follows from Lemma 2 (i) that

(14) $$\begin{array}{c} \frac{h_{\lambda ,\theta }\left(t\right)-h_{\lambda ,\theta }\left(0\right)}{t}<h_{\lambda ,\theta }^{\prime }\left(t\right)\leq h_{0,0}^{\prime }\left(t\right),\;t\in \left(0,R\right). \end{array}$$

Since *h*_*λ*,*θ*_(0) = 0 and *h*_0,0_′(*t*) < 0 for all *t* ∈ (0, *ξ*), we obtain the desired inequality by (14).

**Lemma 4 .**

The constant *ρ* defined in (12) is positive. As a consequence, |*t* − *h*_*λ*,*θ*_(*t*)/*h*_0,0_′(*t*)| < *t* for all *t* ∈ (0, *ρ*).

**Proof**

On one hand, by Lemma 3, it is clear that *h*_*λ*,*θ*_(*t*)/*th*_0,0_′(*t*) − 1 > 0 for *t* ∈ (0, *ξ*). On the other hand, we can obtain from Lemma 2 (i) that

(15) $$\begin{array}{c} \underset{t\to 0}{\lim}\left(\frac{h_{\lambda ,\theta }\left(t\right)}{th_{0,0}^{\prime }\left(t\right)}-1\right)=0. \end{array}$$

Then, we conclude that there exists a *δ* > 0 such that

(16) $$\begin{array}{c} 0<\frac{h_{\lambda ,\theta }\left(t\right)}{th_{0,0}^{\prime }\left(t\right)}-1<1,\;t\in \left(0,\xi \right). \end{array}$$

Therefore, *ρ* is positive. This completes the proof.

Let

(17) $$\begin{array}{c} r≔\min\left\{\rho ,\sigma \right\}, \end{array}$$

where *ρ* and *σ* are given in (12) and (13), respectively. For any starting point **x**_0_ ∈ **B**(**ζ**, *r*)∖{**ζ**}, let {*t*_*k*_} denote the sequence generated by

(18) $$\begin{array}{c} t_{0}=||x_{0}-\zeta ||,\;\;t_{k+1}=\left|t_{k}-\frac{h_{\lambda ,\theta }\left(t_{k}\right)}{h_{0,0}^{\prime }\left(t_{k}\right)}\right|,\;k=0,1,2,\ldots . \end{array}$$

**Lemma 5 .**

The sequence {*t*_*k*_} given by (18) is well defined, strictly decreasing, and contained in (0, *ρ*) and converges to 0.

**Proof**

Since 0 < *t*_0_ = ||**x**_0_ − **ζ**||<*r* ≤ *ρ*, using Lemma 4, one has that {*t*_*k*_} is well defined, strictly decreasing, and contained in [0, *ρ*). Thus, there exists t* ∈ [0, *ρ*) such that lim⁡_*k*→*∞*_ *t*_*k*_ = t*. That is to say, we have

(19) $$\begin{array}{c} 0\leq t^{*}=\frac{h_{\lambda ,\theta }\left(t^{*}\right)}{h_{0,0}^{\prime }\left(t^{*}\right)}-t^{*}<\rho . \end{array}$$

If t* ≠ 0, it follows from Lemma 4 that

(20) $$\begin{array}{c} \frac{h_{\lambda ,\theta }\left(t^{*}\right)}{h_{0,0}^{\prime }\left(t^{*}\right)}-t^{*}<t^{*}. \end{array}$$

This is a contradiction. So *t*_*k*_ → 0 as *k* → *∞*. This completes the proof.

Based on the researches in [7, 14, 18, 20] for the convergence of various Newton-type methods, we present the following modified majorant condition.

**Definition 6 .**

Let *r* > 0 be such that **B**(**ζ**, *r*) ⊂ *Ω*. Then, *DF* is said to satisfy the majorant condition on **B**(**ζ**, *r*) if

(21) ||DF(ζ)†[DF(x)−DF(ζ+τ(x−ζ))]|| ≤hλ,θ′(||x−ζ||)−hλ,θ′(τ||x−ζ||)

for any **x** ∈ **B**(**ζ**, *r*) and *τ* ∈ [0,1].

In the case when *DF*(**ζ**) is not surjective (see [9, 10]), the information on im *DF*(**ζ**)^⊥^ may be lost. To this end, we need to modify the above notion to suit the case when *DF*(**ζ**) is not surjective.

**Definition 7 .**

Let *r* > 0 be such that **B**(**ζ**, *r*) ⊂ *Ω*. Then, *f*′ is said to satisfy the modified majorant condition on **B**(**ζ**, *r*) if

(22) ||DF(ζ)†||||DF(x)−DF(ζ+τ(x−ζ))|| ≤hλ,θ′(||x−ζ||)−hλ,θ′(τ||x−ζ||)

for any **x** ∈ **B**(**ζ**, *r*) and *τ* ∈ [0,1].

## 3. Main Results

In this section, we state our main results of local convergence for inexact Newton method (2). Recall that (1) is a surjective-underdetermined (resp., injective-overdetermined) system if the number of equations is less (resp., greater) than the number of unknowns and *DF*(**x**) is of full rank for each **x** ∈ *Ω*. Note that, for surjective-underdetermined systems, the fixed points of the Newton operator *N*_*F*_(**x**)∶ = **x** − *DF*(**x**)^†^ *F*(**x**) are the zeros of *F*, while for injective-overdetermined systems, the fixed points of *N*_*F*_ are the least square solutions of (1), which, in general, are not necessarily the zeros of *F*.

Our first result concerns the local convergence properties of inexact Newton's method for general singular systems with constant rank derivatives.

**Theorem 8 .**

Let *F* : *Ω* ⊂ *R* ^*n*^ → *R* ^*m*^ be continuously Fréchet differentiable nonlinear operator, where *Ω* is open and convex. Suppose that *F*(**ζ**) = 0, *DF*(**ζ**) ≠ 0 and that *DF* satisfies the modified majorant condition (22) on **B**(**ζ**, *r*), where *r* is given in (17). In addition, we assume that rank *DF*(**x**) ≤ rank *DF*(**ζ**) for any **x** ∈ **B**(**ζ**, *r*) and that

(23) $$\begin{array}{c} ||\left[I_{R^{n}}-DF{\left(x\right)}^{†}DF\left(x\right)\right]\left(x-\zeta \right)||\leq \theta ||x-\zeta ||,\;x\in B\left(\zeta ,r\right), \end{array}$$

where the constant *θ* satisfies 0 ≤ *θ* < 1. Let {**x**_*k*_} be sequence generated by inexact Newton's method with any initial point **x**_0_ ∈ **B**(**ζ**, *r*)∖{**ζ**} and the conditions for the residual **r**_*k*_ and the forcing term *λ*_*k*_:

(24) ||rk||≤λk||F(xk)||,  0≤λkκ(DF(xk))≤λ,k=0,1,2,…,

where *κ*(*A*): = ||*A* ^†^||||*A*|| denotes the condition number of *A* ∈ *R* ^*m*×*n*^. Then, {**x**_*k*_} converges to a zero **ζ** of *DF*(·)^†^ *F*(·) in $\bar{B(\zeta ,r)}$. Moreover, we have the following estimate:

(25) $$\begin{array}{c} ||x_{k+1}-\zeta ||\leq \frac{t_{k+1}}{t_{k}}||x_{k}-\zeta ||,\;k=0,1,2,\ldots , \end{array}$$

where the sequence {*t*_*k*_} is defined by (18).

**Remark 9 .**

If *h*_*λ*,*θ*_(*t*) is given by

(26) $$\begin{array}{c} h_{\lambda ,\theta }\left(t\right)=-\left(1+\lambda +\theta \right)t+\overset{t}{\underset{0}{\int }}L\left(u\right)\left(t-u\right)\;du,\;t\in \left[0,R\right], \end{array}$$

then the results obtained in Theorem 8 reduce to the one given in [19]. Moreover, if taking *λ* = 0 (in this case *λ*_*k*_ = 0 and **r**_*k*_ = 0) in Theorem 8, we obtain the local convergence of Newton's method for solving the singular systems, which has been studied by Dedieu and Kim in [10] for analytic singular systems with constant rank derivatives and Li et al. in [13] for some special singular systems with constant rank derivatives.

If *DF*(**x**) is full column rank for every **x** ∈ **B**(**ζ**, *r*), then we have *DF*(**x**)^†^ *DF*(**x**) = **I**_*R*^*n*^_. Thus,

(27) $$\begin{array}{c} ||\left[I_{R^{n}}-DF{\left(x\right)}^{†}DF\left(x\right)\right]\left(x-\zeta \right)||=0. \end{array}$$

That is, *θ* = 0. We immediately have the following corollary.

**Corollary 10 .**

Suppose that rank *DF*(**x**) ≤ rank *DF*(**ζ**) and that

(28) $$\begin{array}{c} ||\left[I_{R^{n}}-DF{\left(x\right)}^{†}DF\left(x\right)\right]\left(x-\zeta \right)||=0 \end{array}$$

for any **x** ∈ **B**(**ζ**, *r*). Suppose that *F*(**ζ**) = 0, *DF*(**ζ**) ≠ 0 and that *DF* satisfies the modifed majorant condition (22). Let {**x**_*k*_} be sequence generated by inexact Newton's method with any initial point **x**_0_ ∈ **B**(**ζ**, *r*)∖{**ζ**} and the condition (24) for the residual **r**_*k*_ and the forcing term *λ*_*k*_. Then, {**x**_*k*_} converges to a zero **ζ** of *DF*(·)^†^ *F*(·) in $\bar{B(\zeta ,r)}$. Moreover, we have the following estimate:

(29) $$\begin{array}{c} ||x_{k+1}-\zeta ||\leq \frac{t_{k+1}}{t_{k}}||x_{k}-\zeta ||,\;k=0,1,2,\ldots , \end{array}$$

where the sequence {*t*_*k*_} is defined by (18) for *θ* = 0.

In the case when *DF*(**ζ**) is full row rank, the modified majorant condition (22) can be replaced by the majorant condition (21).

**Theorem 11 .**

Suppose that *F*(**ζ**) = 0 and *DF*(**ζ**) is full row rank and that *DF* satisfies the majorant condition (21) on **B**(**ζ**, *r*), where *r* is given in (17). In addition, we assume that rank *DF*(**x**) ≤ rank *DF*(**ζ**) for any **x** ∈ **B**(**ζ**, *r*) and that condition (23) holds. Let {**x**_*k*_} be sequence generated by inexact Newton's method with any initial point **x**_0_ ∈ **B**(**ζ**, *r*)∖{**ζ**} and the conditions for the residual **r**_*k*_ and the forcing term *λ*_*k*_:

(30) ||DF(ζ)†rk||≤λk||DF(ζ)†F(xk)||,0≤λkκ(DF(ζ)†DF(xk))≤λ,k=0,1,2,….

Then, {**x**_*k*_} converges to a zero **ζ** of *F*(·) in $\bar{B(\zeta ,r)}$. Moreover, we have the following estimate:

(31) $$\begin{array}{c} ||x_{k+1}-\zeta ||\leq \frac{t_{k+1}}{t_{k}}||x_{k}-\zeta ||,\;k=0,1,2,\ldots , \end{array}$$

where the sequence {*t*_*k*_} is defined by (18).

**Remark 12 .**

When *h*_*λ*,*θ*_(*t*) is given by (26), the results obtained in Theorem 11 reduce to the ones given in [19].

**Theorem 13 .**

Suppose that *F*(**ζ**) = 0, *DF*(**ζ**) is full row rank and that *DF* satisfies the majorant condition (21) on **B**(**ζ**, *r*), where *r* is given in (17). In addition, we assume that rank *DF*(**x**) ≤ rank *DF*(**ζ**) for any **x** ∈ **B**(**ζ**, *r*) and that condition (23) holds. Let {**x**_*k*_} be sequence generated by inexact Newton method with any initial point **x**_0_ ∈ **B**(**ζ**, *r*)∖{**ζ**} and the conditions for the control residual **r**_*k*_ and the forcing term *λ*_*k*_:

(32) ||DF(xk)†rk||≤λk||DF(xk)†F(xk)||,0≤λkκ(DF(xk))≤λ,k=0,1,2,….

Then, {**x**_*k*_} converges to a zero **ζ** of *f*(·) in $\bar{B(\zeta ,r)}$. Moreover, we have the following estimate:

(33) $$\begin{array}{c} ||x_{k+1}-\zeta ||\leq \frac{t_{k+1}}{t_{k}}||x_{k}-\zeta ||,\;k=0,1,2,\ldots , \end{array}$$

where the sequence {*t*_*k*_} is defined by (18).

**Remark 14 .**

In the case when *DF*(**ζ**) is invertible in Theorem 13 and *h*_*λ*,*θ*_ is given by (26), we obtain the local convergence results of inexact Newton method for nonsingular systems, and the convergence ball *r* in this case satisfies

(34) $$\begin{array}{c} \frac{\int_{0}^{r}L\left(u\right)u\;du}{r\left(\left(1-\lambda \right)-\int_{0}^{r}L\left(u\right)\;du\right)}\leq 1,\;\lambda \in \left[0,1\right). \end{array}$$

In particular, if taking *λ* = 0, the convergence ball *r* determined in (34) reduces to the one given in [6] by Wang and the value *r* is the optimal radius of the convergence ball when the equality holds. Now, Theorem 13 merges into the theory of Newton's method obtained in [6].

**Example 15 .**

Let *R* ^2^ be endowed with the *l*_1_-norm. Consider the operator *F* : *R* ^2^ → *R* ^2^ defined by

(35) $$\begin{array}{c} F\left(x\right)∶=\left[\begin{array}{c} \sin\left(x_{1}-x_{2}\right)-\frac{1}{2}\\ \cos\left(x_{1}-x_{2}\right)-\frac{\sqrt{3}}{2} \end{array}\right],\;\forall x=\left[\begin{array}{c} x_{1}\\ x_{2} \end{array}\right]\in R^{2}. \end{array}$$

Then, *F* is analytic on *R* ^2^ and its derivative is given by

(36) DF(x)=[cos⁡⁡(x1−x2)        −cos⁡⁡(x1−x2)−sin⁡(x1−x2)sin⁡(x1−x2)],∀x=[x1x2]∈R2.

Clearly, rank⁡ (*DF*(**x**)) = 1 and the Moore-Penrose inverse is

(37) DF(x)†=12[cos⁡⁡(x1−x2)        −sin⁡(x1−x2)−cos⁡⁡(x1−x2)sin⁡(x1−x2)],∀x=[x1x2]∈R2.

Moreover, by mathematical induction, for *k* = 1,2,…, we can obtain that

(38) DkF(x)u1⋯uk=12[sin(x1−x2+kπ2)cos⁡(x1−x2+kπ2)]∏i=1k(ui1−ui2),ui=[ui1ui2]∈R2, i=1,…,k.

So, we have

(39) $$\begin{array}{c} ||DF{\left(x\right)}^{†}||=\max\left\{\left|\cos\left(x_{1}-x_{2}\right)\right|,\left|\sin\left(x_{1}-x_{2}\right)\right|\right\},\\ ||D^{k}F\left(x\right)||=\left|\cos\left(x_{1}-x_{2}\right)\right|+\left|\sin\left(x_{1}-x_{2}\right)\right|. \end{array}$$

Consequently,

(40) γ(F,x)∶= sup⁡k≥2(||DF(x)†||||DkF(x)k!||)1/(k−1)=|cos⁡(x1−x2)|+|sin(x1−x2)|2 ×max⁡{|cos⁡(x1−x2)|,|sin(x1−x2)|}.

Let

(41) $$\begin{array}{c} \Omega ∶=\left\{{\left(x_{1},x_{2}\right)}^{T}:\frac{\pi }{5}<x_{1}<\frac{3\pi }{10},\;\;0<x_{2}<\frac{\pi }{10}\right\}\subset R^{2}. \end{array}$$

Then, for any (*x*_1_, *x*_2_)^*T*^ ∈ *Ω*, we have

(42) $$\begin{array}{c} \frac{\sqrt{5}-1}{4}<\sin\left(x_{1}-x_{2}\right)<\frac{\sqrt{5}+1}{4},\\ \sqrt{\frac{5}{8}-\frac{\sqrt{5}}{8}}<\cos\left(x_{1}-x_{2}\right)<\sqrt{\frac{5}{8}+\frac{\sqrt{5}}{8}}. \end{array}$$

Consider the point **ζ** = (13*π*/60, *π*/20)^*T*^. Then, **ζ** ∈ *Ω* satisfies *F*(**ζ**) = 0 and

(43) $$\begin{array}{c} \gamma ∶=\gamma \left(F,\zeta \right)=\frac{3+\sqrt{3}}{8}. \end{array}$$

Hence, we have

(44) ||[IRn−DF(x)†DF(x)](x−ζ)||≤(58+58)||x−ζ||,x∈Ω.

That is, (23) holds with $\theta =5/8+\sqrt{5}/8<1$. Furthermore, it is clear that

(45) $$\begin{array}{c} \kappa \left(DF\left(x_{k}\right)\right)=||DF\left(x_{k}\right)||||DF{\left(x\right)}^{†}||=2\left(\frac{5}{8}+\frac{\sqrt{5}}{8}\right). \end{array}$$

So, we can choose the forcing term {*λ*_*k*_} such that 0 ≤ *λ*_*k*_ ≤ *λ*_max⁡_ = 1/2; see [23] for more details. Then (24) holds with $\lambda =5/8+\sqrt{5}/8$. Now, the majorizing function *h*_*λ*,*θ*_(*t*) associated with the operator *F* defined by (35) reduces to

(46) hλ,θ(t) =−(1+(58+58)+(58+58))t  +((3+3)/8)t21−((3+3)/8)t =−9+54t+((3+3)/8)t21−((3+3)/8)t,t∈[0,4(3−3)3).

Then we have the convergence radius *r* = 0.0604146…. Therefore, Theorem 8 is applicable to concluding that, for any **x**_0_ ∈ **B**(**ζ**, *r*), the sequence {**x**_*k*_} generated by inexact Newton method (2) with initial point **x**_0_ converges to a zero of *DF*(·)^†^ *F*(·).

## 4. Proofs

In this section, we prove our main results of local convergence for inexact Newton method (2) given in Section 3.

### 4.1. Proof of Theorem 8

**Lemma 16 .**

Suppose that *DF* satisfies the modified majorant condition on **B**(**ζ**, *r*) and that ||**ζ** − **x**||<min⁡{*ρ*, *ξ*}, where *r*, *ρ*, and *ξ* are defined in (17), (12), and (11), respectively. Then, rank *DF*(**x**) = rank *DF*(**ζ**) and

(47) $$\begin{array}{c} ||\text{D}F{\left(x\right)}^{†}||\leq -\frac{||\text{D}F{\left(\zeta \right)}^{†}||}{h_{0,0}^{\prime }\left(||x-\zeta ||\right)}. \end{array}$$

**Proof**

Since *h*_0,0_′(0) = −1, we have

(48) ||DF(ζ)†||||DF(x)−DF(ζ)||≤h0,0′(||x−ζ||)−h0,0′(0)<−h0,0′(0)=1.

It follows from Lemma 1 that rank⁡ *DF*(**x**) = rank⁡ *DF*(**ζ**) and

(49) $$\begin{array}{c} ||DF{\left(x\right)}^{†}||\leq \frac{||DF{\left(\zeta \right)}^{†}||}{1-\left(h_{0,0}^{\prime }\left(||x-\zeta ||\right)-h_{0,0}^{\prime }\left(0\right)\right)}=-\frac{||DF{\left(\zeta \right)}^{†}||}{h_{0,0}^{\prime }\left(||x-\zeta ||\right)}. \end{array}$$

This completes the proof.

**Proof of Theorem 8.**

We will prove by induction that {*t*_*k*_} is the majorizing sequence for {**x**_*k*_}; that is,

(50) $$\begin{array}{c} ||\zeta -x_{j}||\leq t_{j}\;\forall j=0,1,2,\ldots . \end{array}$$

Because *t*_0_ = ||**x**_0_ − **ζ**||, (50) holds for *j* = 0. Now, we assume that ||**ζ** − **x**_*j*_||≤*t*_*j*_ for some *j* = *k* ∈ *N*. For the case *j* = *k* + 1, we first notice that

(51) xk+1−ζ =xk−ζ−DF(xk)†[F(xk)−F(ζ)]+DF(xk)†rk =DF(xk)†[F(ζ)−F(xk)−DF(xk)(ζ−xk)]  +DF(xk)†rk+[IRn−DF(xk)†DF(xk)](xk−ζ) =DF(xk)†∫01[DF(xk)−DF(ζ+τ(xk−ζ))](xk−ζ)dτ  +DF(xk)†rk+[IRn−DF(xk)†DF(xk)](xk−ζ).

By using the modified majorant condition (22), Lemma 16, the inductive hypothesis (50), and Lemma 2, one has that

(52) ||DF(xk)†∫01[DF(xk)−DF(ζ+τ(xk−ζ))](xk−ζ)dτ|| ≤−1h0,0′(||xk−ζ||)∫01||DF(ζ)†||  ×||DF(xk)−DF(ζ+τ(xk−ζ))||  ×||xk−ζ||dτ =−1h0,0′(||xk−ζ||)  ×∫01hλ,0′(||xk−ζ||)−hλ,0′(τ||xk−ζ||)||xk−ζ||dτ·||xk−ζ||2 ≤−1h0,0′(tk)∫01hλ,0′(tk)−hλ,0′(τtk)tkdτ·||xk−ζ||2 =−1h0,0′(tk)(tkhλ,0′(tk)−hλ,0(tk))||xk−ζ||2tk2.

Thanks to (24),

(53) $$\begin{array}{c} ||DF{\left(x_{k}\right)}^{†}r_{k}||\leq ||DF{\left(x_{k}\right)}^{†}||||r_{k}||\leq \lambda_{k}||DF{\left(x_{k}\right)}^{†}||||F\left(x_{k}\right)||. \end{array}$$

Since

(54) −F(xk) =F(ζ)−F(xk)−DF(xk)(ζ−xk)+DF(xk)(ζ−xk) =∫01[DF(xk)−DF(ζ+τ(xk−ζ))](xk−ζ)dτ  +DF(xk)(ζ−xk),

combining Lemma 2, Lemma 16, the modified majorant condition (22), the inductive hypothesis (50), and the condition (24), we have

(55) λk||DF(xk)†||||F(xk)|| ≤λk||DF(xk)†||  ×∫01||DF(xk)−DF(ζ+τ(xk−ζ))||    ×||xk−ζ||dτ    +λk||DF(xk)†||||DF(xk)||||xk−ζ|| ≤−λh0,0′(tk)(tkhλ,0′(tk)−hλ,0(tk))||xk−ζ||2tk2  +λtk·||xk−ζ||tk≤λλtk+hλ,0(tk)h0,0′(tk)·||xk−ζ||tk.

Combining (23), (52), (53), and (55), we can obtain that

(56) ||xk+1−ζ|| ≤[−tkhλ,0′(tk)−hλ,0(tk)h0,0′(tk)+λ·λtk+hλ,0(tk)h0,0′(tk)+θtk]||xk−ζ||tk =[−tk+(1+λ)(λtkh0,0′(tk)+hλ,0(tk)h0,0′(tk))+θtk]||xk−ζ||tk.

Note that −1 < *h*_0,0_′(*t*) < 0 for any *t* ∈ (0, *ρ*),

(57) (1+λ)(λtkh0,0′(tk)+hλ,0(tk)h0,0′(tk))+θtk≤hλ,0(tk)h0,0′(tk)  +θtk≤hλ,0(tk)−θtkh0,0′(tk)=hλ,θ(tk)h0,0′(tk).

Thus, in view of the definition of {*t*_*k*_} given in (18), one has that

(58) $$\begin{array}{c} ||x_{k+1}-\zeta ||\leq \frac{t_{k+1}}{t_{k}}||x_{k}-\zeta ||, \end{array}$$

which implies that ||**x**_*k*+1_ − **ζ**||≤*t*_*k*+1_. Therefore, the proof by induction is complete. Since {*t*_*k*_} converges to 0 (by Lemma 5), it follows from (50) that {**x**_*k*_} converges to **ζ** and the estimate (25) holds for all *k* ≥ 0. This completes the proof.

### 4.2. Proof of Theorem 11

**Lemma 17 .**

Suppose that *F*(**ζ**) = 0, *DF*(**ζ**) is full row rank, and *DF* satisfies the majorant condition (21) on **B**(**ζ**, *r*). Then, for any **x** ∈ **B**(**ζ**, *r*), we have rank *DF*(**x**) = rank *DF*(**ζ**) and

(59) $$\begin{array}{c} ||{\left[I_{R^{n}}-DF{\left(\zeta \right)}^{†}\left(DF\left(\zeta \right)-DF\left(x\right)\right)\right]}^{-1}||\leq -\frac{1}{h_{0,0}^{\prime }\left(||x-\zeta ||\right)}. \end{array}$$

**Proof**

Since *h*_0,0_′(0) = −1, we have

(60) ||DF(ζ)†[DF(x)−DF(ζ)]|| ≤h0,0′(||x−ζ||)−h0,0′(0)<−h0,0′(0)=1.

It follows from Banach lemma that [**I**_*R*^*n*^_ − *DF*(**ζ**)^†^(*DF*(**ζ**) − *DF*(**x**))]^−1^ exists and

(61) $$\begin{array}{c} ||{\left[I_{R^{n}}-DF{\left(\zeta \right)}^{†}\left(DF\left(\zeta \right)-DF\left(x\right)\right)\right]}^{-1}||\leq -\frac{1}{h_{0,0}^{\prime }\left(||x-\zeta ||\right)}. \end{array}$$

Since *DF*(**ζ**) is full row rank, we have *DF*(**ζ**)*DF*(**ζ**)^†^ = **I**_*R*^*m*^_ and

(62) $$\begin{array}{c} DF\left(x\right)=DF\left(\zeta \right)\left[I_{R^{n}}-DF{\left(\zeta \right)}^{†}\left(DF\left(\zeta \right)-DF\left(x\right)\right)\right], \end{array}$$

which implies that *DF*(**x**) is full row rank; that is, rank⁡ *DF*(**x**) = rank⁡ *DF*(**ζ**). The proof is complete.

**Proof of Theorem 11.**

Let $\hat{F}:B(\zeta ,r)\to R^{m}$ be defined by

(63) $$\begin{array}{c} \hat{F}\left(x\right)=DF{\left(\zeta \right)}^{†}F\left(x\right),\;x\in B\left(\zeta ,r\right), \end{array}$$

with residual ${\hat{r}}_{k}=DF{\left(\zeta \right)}^{†}r_{k}$. Since

(64) DF^(x)†=[DF(ζ)†DF(x)]†=DF(x)†DF(ζ),x∈B(ζ,r),

one has that {**x**_*k*_} coincides with the sequence generated by inexact Newton's method (2) for $\hat{F}$. In addition, we have

(65) $$\begin{array}{c} D\hat{F}{\left(\zeta \right)}^{†}={\left(DF{\left(\zeta \right)}^{†}DF\left(\zeta \right)\right)}^{†}=DF{\left(\zeta \right)}^{†}DF\left(\zeta \right), \end{array}$$

and so

(66) ||DF^(ζ)†DF^(ζ)||=||DF(ζ)†DF(ζ)DF(ζ)†F(ζ)||=||DF(ζ)†F(ζ)||.

Because ||*DF*(**ζ**)^†^ *F*(**ζ**)|| = ||Π_*ker*⁡*DF*(**ζ**)^⊥^_|| = 1, we have

(67) $$\begin{array}{c} ||D\hat{F}{\left(\zeta \right)}^{†}||=||D\hat{F}{\left(\zeta \right)}^{†}D\hat{F}\left(\zeta \right)||=1. \end{array}$$

Therefore, by (21), we can obtain that

(68) ||DF^(ζ)†||||DF^(x)−DF^(ζ+τ(x−ζ))|| =||DF(ζ)†(DF(x)−DF(ζ+τ(x−ζ)))|| ≤hλ,θ′(||x−ζ||)−hλ,θ′(τ||x−ζ||).

That is, $\hat{F}$ satisfies the modified majorant condition (22) on **B**(**ζ**, *r*). So, Theorem 8 is applicable and {**x**_*k*_} converges to **ζ** follows. Note that $D\hat{F}{\left(\cdot \right)}^{†}\hat{F}(\cdot )=DF{\left(\cdot \right)}^{†}F(\cdot )$ and *F*(·) = *DF*(·)*DF*(·)^†^ *F*(·); it follows that **ζ** is a zero of *F*.

### 4.3. Proof of Theorem 13

**Lemma 18 .**

Suppose that *F*(**ζ**) = 0, *DF*(**ζ**) is full row rank, and *DF* satisfies the majorant condition (21) on **B**(**ζ**, *r*). Then we have

(69) $$\begin{array}{c} ||DF{\left(x\right)}^{†}DF\left(\zeta \right)||\leq -\frac{1}{h_{0,0}^{\prime }\left(||x-\zeta ||\right)},\;x\in B\left(\zeta ,r\right). \end{array}$$

**Proof**

Since *DF*(**ζ**) is full row rank, we have *DF*(**ζ**)*DF*(**ζ**)^†^ = **I**_*R*^*m*^_. It follows that

(70) DF(x)†DF(ζ)(IRn−DF(ζ)†(DF(ζ)−DF(x))) =DF(x)†DF(x), x∈B(ζ,r).

By Lemma 17, **I**_*R*^*n*^_ − *DF*(**ζ**)^†^(*DF*(**ζ**) − *DF*(**x**)) is invertible for any **x** ∈ **B**(**ζ**, *r*). Thus, in view of the equality *A* ^†^ *A* = Π_*ker*⁡*A*^⊥^_ for any *m* × *n* matrix *A*, one has that

(71) DF(x)†DF(ζ) =Πker⁡DF(x)⊥[IRn−DF(ζ)†(DF(ζ)−DF(x))]−1.

Therefore, Lemma 17 is applicable to conclude that

(72) ||DF(x)†DF(ζ)|| ≤||Πker⁡DF(x)⊥||||[IRn−DF(ζ)†(DF(ζ)−DF(x))]−1|| ≤−1h0,0′(||x−ζ||).

The proof is complete.

**Proof of Theorem 13.**

Using Lemma 18, majorant condition (21), and the residual condition (32), respectively, instead of Lemma 16, modified majorant condition (22), and condition (24), one can complete the proof of Theorem 13 as the same line of proof in Theorem 8.
